# Obesity-associated up-regulation of lipocalin 2 protects gastric mucosa cells from apoptotic cell death by reducing endoplasmic reticulum stress

**DOI:** 10.1038/s41419-021-03512-2

**Published:** 2021-02-26

**Authors:** Xin Wen, Bin Su, Mingming Gao, Jiaqi Chen, Donglei Zhou, Hui You, Nannan Li, Shuaikang Chang, Xiaoyun Cheng, Chunhua Qian, Jingyang Gao, Peng Yang, Shen Qu, Le Bu

**Affiliations:** 1 Department of Endocrinology and Metabolism, Shanghai Tenth People’s Hospital, Tongji University School of Medicine, Shanghai, 200072 China; 2 National Metabolic Management Center, Shanghai, 200072 China; 3 Department of Pharmaceutical and Biomedical Sciences, College of Pharmacy, University of Georgia, 250 West Green Street, Athens, GA 30602 USA; 4 Department of Endocrinology and Metabolism, Suzhou Municipal Hospital, The Affiliated Suzhou Hospital of Nanjing Medical University, Suzhou, China; 5 Department of Gastrointestinal Surgery, Shanghai Tenth People’s Hospital, Tongji University School of Medicine, Shanghai, 200072 China; 6 Department of Hematology, Shanghai Tenth People’s Hospital, Tongji University School of Medicine, Shanghai, 200072 China

**Keywords:** Apoptosis, Stomach diseases

## Abstract

Gastric mucosal injury is a less well known complication of obesity. Its mechanism remains to be further elucidated. Here, we explored the protective role of lipocalin 2 (LCN2) against endoplasmic reticulum stress and cell apoptosis in gastric mucosa in patients and mice with obesity. Through molecular and genetic analyses in clinical species, LCN2 secreted by parietal cells expression is elevated in obese. Immunofluorescence, TUNEL, and colorimetry results show that a more significant upregulation of pro-inflammatory factors and increased amount of apoptotic cells in gastric tissue sections in obese groups. Loss- and gain-of-function experiments in gastric epithelial cells demonstrate that increased LCN2 protected against obesity associated gastric injury by inhibiting apoptosis and improving inflammatory state. In addition, this protective effect was mediated by repressing ER stress. Our findings identify LCN2 as a gastric hormone could be a compensatory protective factor against gastric injury in obese.

## Introduction

Obesity is now becoming a worldwide serious problem. Obesity is associated with a spectrum of severe diseases, including diabetes, cardiovascular diseases, and cancers. Also, gastrointestinal diseases are complications of obesity. In the earlier study^1^, transcriptomic analysis of different parts of the GI tract (stomach, duodenum, jejunum, ileum, ascending colon, and descending colon) found that the stomach was the tissue with the most differentially regulated genes in the high fat diet-induced model. Obese patients have a higher prevalence of chronic gastric infection, gastritis, peptic ulcer and gastric adenocarcinoma^2,3^. Animal studies also find that dietary lipids can induce parietal cell damage and may lead to precancerous metaplasia^4^.

Lipocalin 2 (LCN2), also known as neutrophil gelatinase associated lipocalin (NGAL), is a member of the lipocalin family. Due to its upregulated expression in infection, LCN2 is originally considered to be a key regulator of immune response^5^. Subsequent investigations revealed that LCN2 is expressed in many tissues and is related to a variety of diseases, including obesity^6^. LCN2 was reported to be increased in obesity individuals^7^. Studies in animal models also elucidated that elevated LCN2 levels were observed in obese mice^8^. LCN2 is highly expressed in many pathological injury conditions such as acute and chronic kidney injury, heart failure, brain injury, inflammatory bowel disease and breast cancer^9^. Both the protective and harmful roles of LCN2 are documented in these injury diseases. Despite these recent studies, the relationship between LCN2 expression and obesity associated gastric injury remains largely unclear.

In the current study, we examined LCN2 expression and found that it was elevated in gastric mucosa in obesity individuals. We further explored the potential functions of LCN2 upregulation in this context and demonstrated increased LCN2 protected against obesity associated gastric injury. Mechanistic studies revealed that this protective effect was mediated by repressing ER stress.

Therefore, the main objective of this study is to investigate whether LCN2 overexpression protects against obesity associated gastric injury and to explore the underlying mechanisms.

## Results

### Alterations of gene expression profiles of gastric mucosa in patients with obesity

**Fig. 1:**
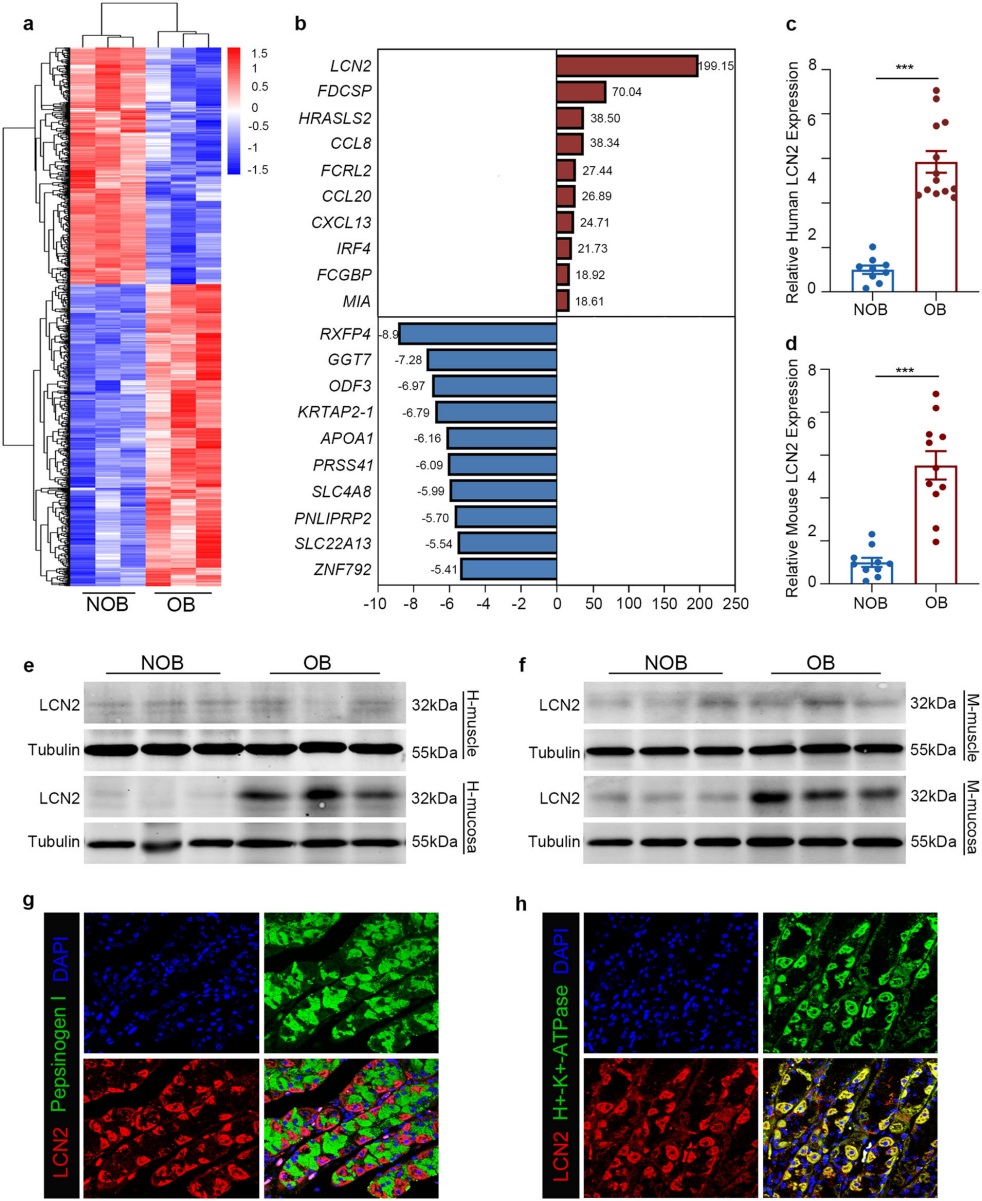
LCN2 levels are elevated in obese group, and LCN2 is localized to parietal cells. **a** Heatmap of differentially expressed genes between obese individuals and normal controls. Red and blue characterize up-regulated and down-regulated genes, respectively. *n* = 3(NOB patients), *n* = 3(OB patients). **b** Top 10 up- and down-regulated genes in microarray analysis. **c, d**, mRNA expression of LCN2 in gastric mucosa of humans (**c**) n = 9(NOB patients), *n* = 13(OB patients) or mice (**d**) *n* = 10(NOB mice), *n* = 11(OB mice). Western blot analysis of LCN2 levels in gastric mucosa and muscle of humans (**e**) or mice (**f**). Tubulin was used as a loading control. Representative immunofluorescence staining shows (**g**) lack of co-localization of LCN2 (red) with marker Pepsinogen I (green) in chief cells, and (**h**) co-localization of LCN2 (red) with marker H^+^-K^+^-ATPase (green) in parietal cells. Data are mean ± s.e.m.; ***P < 0.005 (Student’s *t* test).

Mounting evidence suggests that obesity is associated with gastric dysfunctions^3,10,11^. In order to study the changes of gastric mucosal genes in obese patients, we extracted the total RNA from gastric mucosa of non-obese and obese patients for microarray gene expression analysis. As summarized in the heat map (Fig. 1a), a total of 929 genes were differentially expressed between the NOB and OB groups (*p* < 0.05, fold change> 2), 531 genes were up-regulated while 397 genes down-regulated in the OB group compared to the NOB group. LCN2 was one of the top genes showing upregulated expression in the stomach of obese patients (Fig. 1b). Despite previous studies^12^, the role of LCN2 upregulation in obesity associated gastric dysfunction remains largely elusive. We therefore focused on the LCN2 gene in the current study.

### LCN2 is a gastrointestinal hormone derived from parietal cells

To verify the high expression of LCN2 in the stomach of the obese group, we measured both mRNA and protein levels of LCN2 in mice and humans (Fig. 1c, d, e, f). In line with the microarray data, our quantitative PCR and western blotting data confirmed that LCN2 was remarkably upregulated in the stomach of both obese animal models (by ~5.52-fold) and human patients (by ~5.85-fold). The next question is which group of cells produces LCN2 in the stomach. To address this question, we performed immunofluorescence to detect LCN2 expression in the gastric mucosal layer. Our immunofluorescence data clearly demonstrated that the LCN2 signal was co-localized with hydrogen potassium ATPase, a marker of parietal cells, but not pepsinogen I, a marker for chief cells and mucus neck cells (Fig. 1g, h). These results revealed that, in the stomach, LCN2 was mainly expressed in parietal cells.

### Gastric mucosa was injured in obese state

**Fig. 2:**
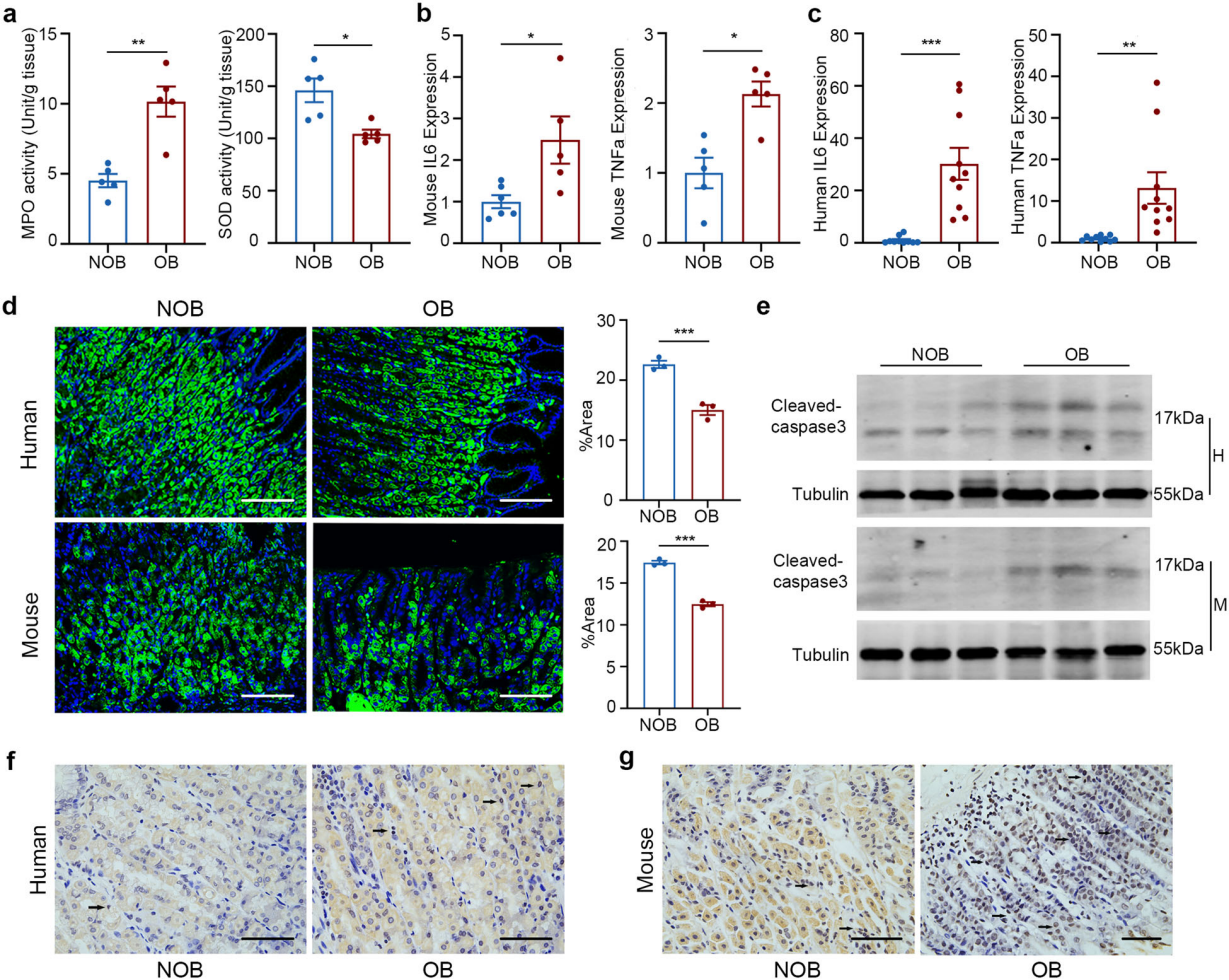
Gastric mucosa was injured in obese group. **a** Increased oxidative stress in obese state. *n* = 5 mice/group. Expression of pro-inflammatory genes in gastric mucosa of mice (**b**) n = 5 mice/group and humans (**c**) *n* = 10 patients/group. **d** Immunofluorescence staining of parietal cells (green) on stomach tissue sections (×10, ×20 magnification; 100 µm scale bar). **e** Western blot analysis of cleaved caspase-3. Tubulin was used as a loading control. Detection of TUNEL-positive cells in stomach tissue in humans (**f**) and mice (**g**) via TUNEL staining, the arrow in the image refers to the apoptotic cells (×40 magnification; 100 µm scale bar).

We next further explored the relationship between obesity and gastric mucosa damage. Previous studies demonstrated that oxidative stress and inflammation lead to gastric injury^13^. Obesity was associated with significantly increased activity of MPO, a marker of neutrophil infiltration into the gastric mucosa (Fig. 2a). We next measured levels of SOD, an enzyme for scavenging oxygen free radicals, and found that the activity of this enzyme was significantly decreased in obese mice (Fig. 2a). Pro-inflammatory factors including TNFα and IL6 were upregulated in obese animal models (Fig. 2b). A more significant upregulation of these pro-inflammatory factors were observed in the stomach of obese human patients (Fig. 2c). Cleaved-caspse3, an apoptotic cell marker, was found to be increased in stomach samples from both obese mice and human patients (Fig. 2e). TUNEL assay further verified increased amount of apoptotic cells in gastric tissue sections (Fig. 2f, g).

Considering LCN2 is secreted primarily from parietal cells, we next further assessed the impact of obesity on parietal cells. Our former study has demonstrated LCN2 is secreted by parietal cells that produce gastric acid. Thus, in the damaged gastric mucosa, whether parietal cells injured accordingly? Immunofluorescence results showed a significant decrease in the number of parietal cells in the gastric mucosa of obese objects (Fig. 2d).

### Acute gastric mucosal injury leads to LCN2 over-expression

**Fig. 3:**
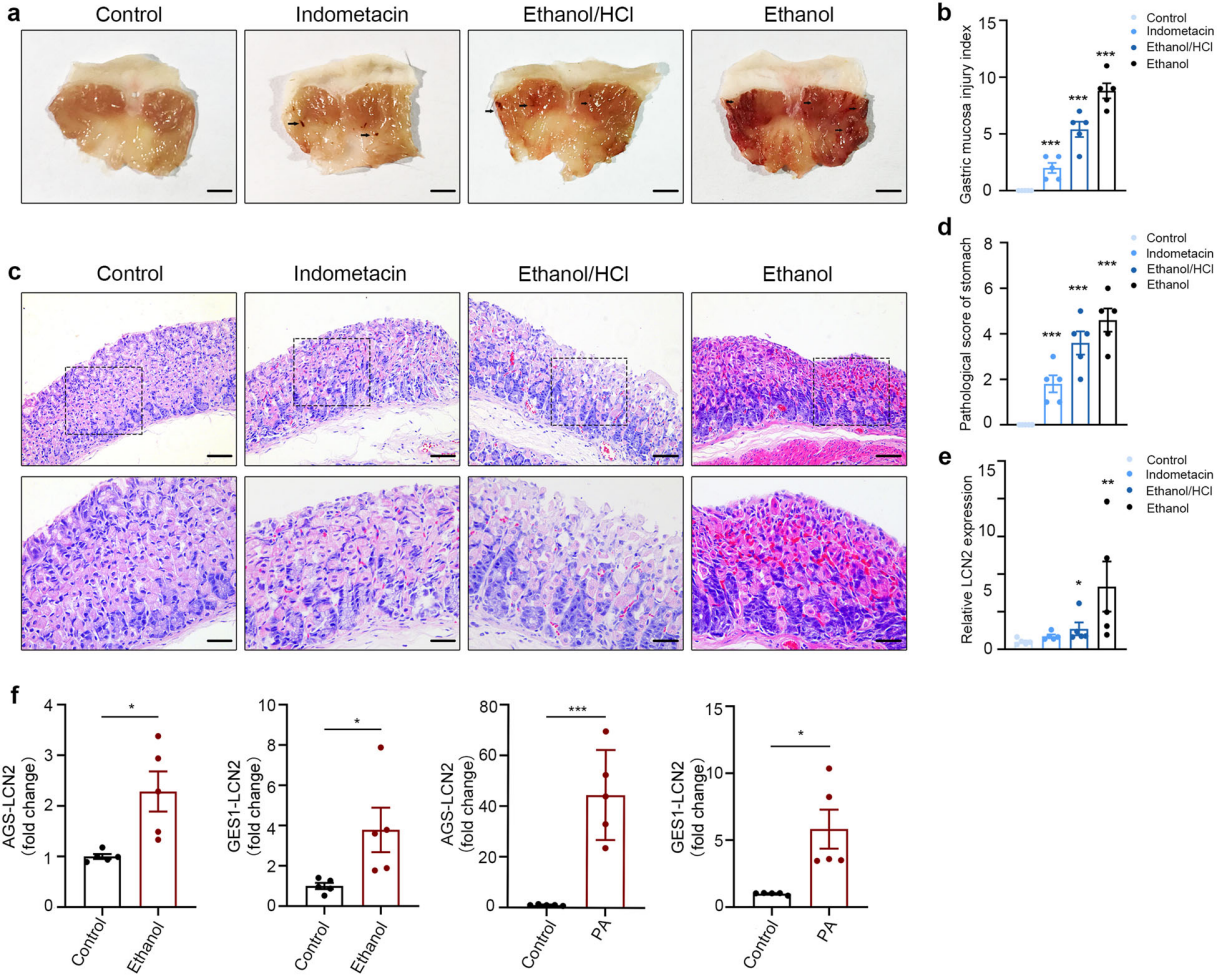
Gastric mucosal injury leads to LCN2 over-expression. **a** Indometacin, acidified ethanol and ethanol induced gastric injured model on the macroscopic properties. Arrows indicate the damage (linear hemorrhages, mucosal erythema and hemorrhagic ulcers) on gastric mucosa. **b** Gastric mucosa injured index of the control, indomethacin, ethanol/HCL and ethanol groups. **c** HE-stained gastric mucosal tissues of mice in four groups (magnification, ×100 and ×200; 100 µm scale bar). **d** The degree of pathological damage in stomach using Masuda criteria. **e** Quantification of LCN2 expression in these four groups. **f** The expression of LCN2 in AGS and GES1 cells simulated with alcohol and palmitic acid. The data are expressed as the mean ± s.e.m. (*n* = 5) *P < 0.05, **P < 0.01, ***P < 0.005 compared to the control group.

LCN2 has been shown as a stress response protein in previous studies^14^, and we have shown that chronic stimuli such as obesity do increase its expression at both gene express and protein levels. In order to further verify that the stimulated gastric mucosa has a higher level of LCN2 gene expression, we used indomethacin, acidified ethanol and ethanol to stimulate the gastric mucosa of mice. Macroscopic evaluation of the gastric mucosa showed that intragastric administration of indomethacin, acidified ethanol and ethanol induced severe gastric mucosal damage, such as linear hemorrhages, mucosal erythema and hemorrhagic ulcers (Fig. 3a). Figure 3b quantified results showed that lesions on the gastric mucosa were significantly increased in those four groups (0 ± 0, 2 ± 0.89, 5.4 ± 1.36, 8.8 ± 1.33, respectively).

Examination of the tissue under light microscopy revealed that indomethacin induced only mild surface injury to oxyntic mucosa of mice, whereas the glandular architecture remained intact. Acidified ethanol induced extensive injury to the gastric mucosa and occasionally involved the muscularis mucosa. Ethanol-induced surface injury to the gastric mucosa and the necrotic ulcerogenic lesions seemed the most severe in mice (Fig. 3c, d). Gene expression analysis revealed that LCN2 was upregulated in the stomach of mice with gastric injuries (Fig. 3e). These results suggested that increased LCN2 expression was associated with mucosal injury in animal models.

In vitro, we treated AGS and GES1 cells with alcohol and palmitic acid (PA) to induce cell damage. Our data showed that the viability of AGS was significantly reduced after treatment with 6% ethanol for 4 h and 400uM PA for 12 h. The viability of GES1 was also significantly decreased after treatment with 4% ethanol for 4 h and 300uM PA for 12 h (Supplementary Fig. 1). Consistent with in vivo results, LCN2, as a stress response protein, was significantly up-regulated after stimulation (Fig. 3f, g).

### LCN2 protects gastric mucosal cells by inhibiting apoptosis

**Fig. 4:**
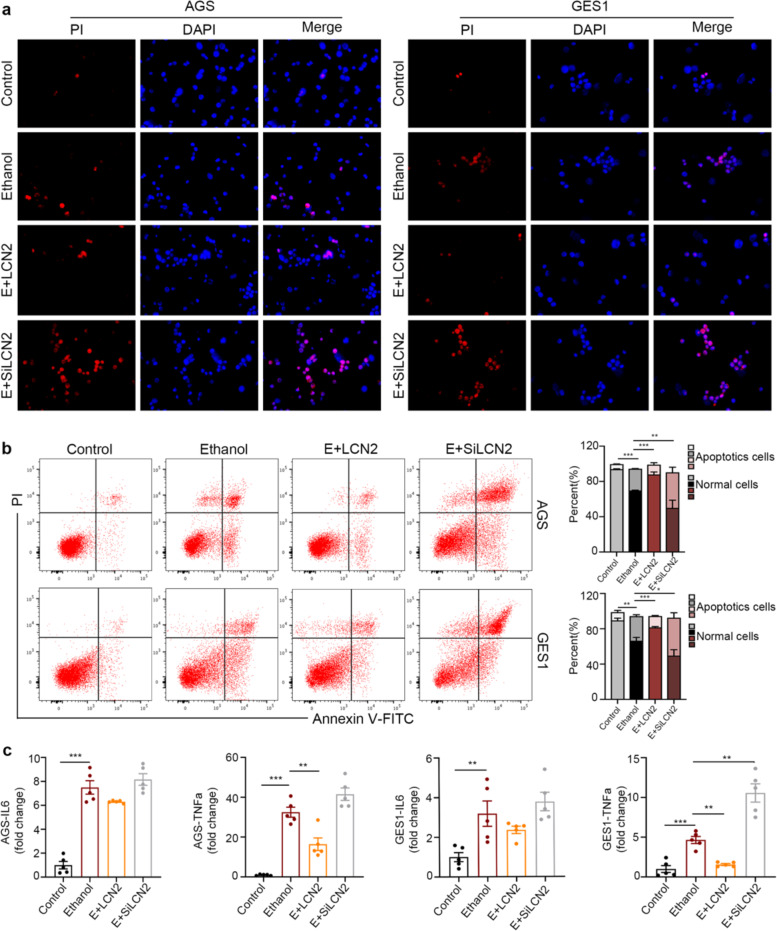
LCN2 protects gastric epithelial cells (AGS, GES1) from ethanol-induced apoptosis and inflammatory state. **a** AGS and CES1 cells groups (NC, LCN2, siRNA-LCN2) were treated with 6% ethanol for 4h, the blank group (Control) was added to the complete medium, and as assayed via staining with DAPI/PI, significant protection against ethanol-induced apoptosis was afforded by LCN2. Channels are: DAPI nuclear stain (blue) and apoptosis by PI (red); ×400 magnification. **b** Cell apoptosis was analyzed by flow cytometry using Annexin V/FITC with the same treatment. Columns (right panel) represent the average percent of Annexin V positive from three independent experiments. **c** The level of IL6 and TNFα in each groups. The data are expressed as the mean ± s.e.m. *P < 0.05, **P < 0.01, ***P < 0.005.

**Fig. 5:**
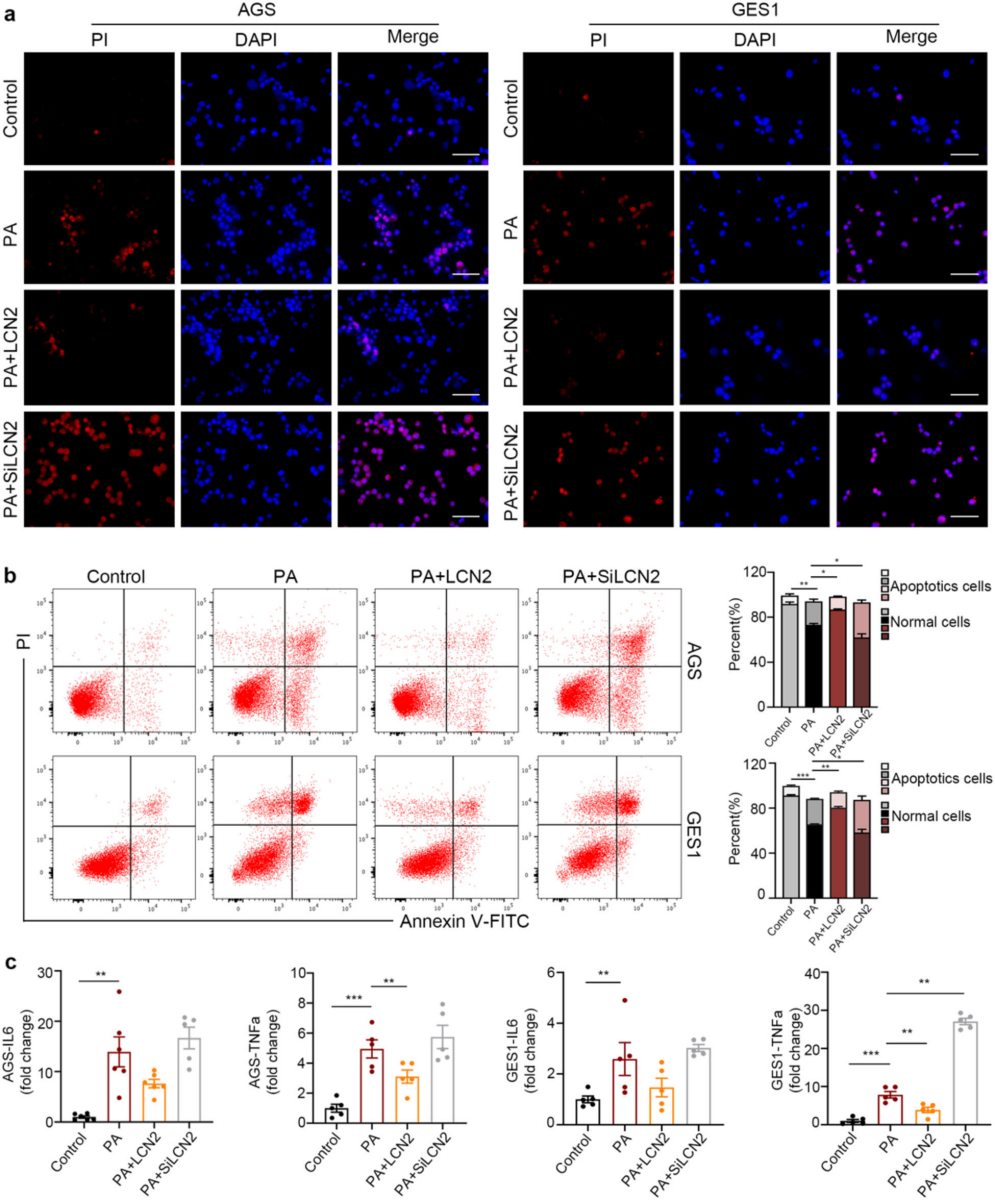
**LCN2 protects gastric epithelial cells (AGS, GES1) from palmitic acid-induced apoptosis and inflammatory state**. **a** AGS and CES1 cells groups (NC, LCN2, siRNA-LCN2) were treated with 300 μM PA for 12h, the blank group (Control) was added to the fatty acid free medium, and as assayed via staining with DAPI/PI, significant protection against PA-induced apoptosis was afforded by LCN2. Channels are: DAPI nuclear stain (blue) and apoptosis by PI (red); ×400 magnification. **b** Cell apoptosis was analyzed by flow cytometry using Annexin V/FITC with the same treatment. Columns (right panel) represent the average percent of Annexin V positive from three independent experiments. **c** The level of IL6 and TNFα in each groups. The data are expressed as the mean ± s.e.m. *P < 0.05, **P < 0.01, ***P < 0.005.

To further investigate the functional role of LCN2 upregulation during gastric mucosal injury, we conducted in vitro studies. As shown in Figs. 4, 5, stimulation with ethanol or PA led to increased cell apoptosis, which was associated with significantly elevated expression of pro-inflammatory factors including TNFα and IL6. Pretreatment with LCN2 recombinant proteins protected cells from cell damage-induced apoptosis and blocked upregulation of pro-inflammatory factors. On the contrary, siRNA knockdown of the LCN2 gene further potentiated cell apoptosis while promoting pro-inflammatory factor gene expression. Taken together, this set findings clearly demonstrated a protective role of overexpressed LCN2 in gastric mucosal injury.

### LCN2 inhibits endoplasmic reticulum stress-mediated apoptosis signaling

**Fig. 6:**
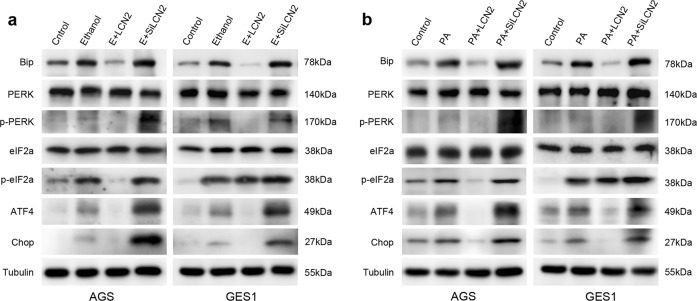
LCN2 inhibits endoplasmic reticulum stress-mediated apoptosis signaling. **a** In ethanol damage model, western blot analysis of phosphorylated (p-) or total PERK, eIF2α and Bip, ATF4, Chop. Tubulin was used as a loading control. **b** In palmitic acid damage model, western blot analysis of phosphorylated (p-) or total PERK, eIF2α and Bip, ATF4, Chop. Tubulin was used as a loading control.

To further investigate the underlying mechanism for this protective effect, we explored main marker proteins involved in ER stress. Western blotting data demonstrated that ER stress pathway was activated by ethanol and PA stimulation in gastric mucosal cells. Exposure to ethanol or PA was associated with overexpression of Bip, phosphor-PERK, phosphor-eIF2a, ATF4, and CHOP (Fig. 6a, b), suggesting activation of cellular ER stress. To determine whether LCN2 is involved in ERS pathway, we treated cells with LCN2 recombinant proteins and knocked down LCN2 using siRNA. LCN2 pretreatment diminished ER stress markers while siRNA knockdown further enhanced expression of these markers, suggesting that LCN2 mitigated cell apoptosis through suppressing ER stress.

## Discussion

In this study, we found that LCN2 expression was increased in the stomach of obese mice and human patients. Increased LCN2 may act as a protective factor in obesity-induced gastric injury by inhibiting endoplasmic reticulum stress-mediated apoptosis.

We firstly investigated LCN2 expression in gastric mucosa of obese individuals by microarray analysis. LCN2 expression was remarkably up-regulated in obese patients compared with non-obese individuals. Western blotting results further confirmed this upregulation in the stomach of obese patients and obese animals. Previous studies demonstrated that LCN2 expression was also elevated in gastric mucosa in *H. Pylori* infection and gastric cancer^15–17^. Our results were in consistent with these studies by showing that LCN2 was mainly expressed and increased in gastric mucosa but not in gastric muscle. However, no further details were given by previous studies about what particular cells were responsible for LCN2 expression. In this study, we located the LCN2 expression by immunofluorescence and clearly demonstrated that it was expressed in parietal cells.

Tissue damage can be identified in a variety of tissues under obesity state. As a barrier, the integrity of gastric mucosa is important to protect deeper located cells^18,19^. Oxidative stress and inflammation are responsible for gastric mucosa injury^13,14,18^. We confirmed gastric injury in obese state by detecting several markers of oxidative stress and some inflammatory cytokines. And we also found that parietal cells were damaged under obesity state. And not surprisingly, gastric LCN2 expression was also upregulated in accompany with increased gastric injury markers.

As reported by former studies, LCN2 is considered as an obesity-related hormone. In obese individuals, serum LCN2 level was reported to be elevated by 60% compared with normal body weight individuals^20^. And another study reported that LCN2 expression was increased in adipose tissue in obese patients^20^. Similarly, in obese mouse models, LCN2 was also increased in the circulation and in the liver and adipose tissues^8,21^. The increase of LCN2 expression may due to inflammation cytokines and fatty acids in obese state. TNF-alpha, IL-1beta, IL-6, and palmitic acid may induce the translation of LCN2 through NF-kappa B pathway^22,23^. Interestingly, LCN2 may in turn alleviate inflammation by regulating NF-kappa B^24^. This feedback loop indicates that the induced LCN2 expression may act as a protective factor in obesity. Also, studies demonstrated that LCN2 may protect against metabolic deterioration such as insulin resistance^25,26^. By treatment of cells with LCN2 recombinant protein or siRNA, we revealed that the elevated LCN2 expression in gastric mucosa in obesity may also be protective. However, the mechanism by which LCN2 alleviates obesity-induced gastric injury is not fully understood.

In both obesity-induced and acute gastric injury, we found gastric mucosa apoptosis level was increased and LCN2 might suppress this process. Apoptosis is a widely studied cell death mechanism related to various diseases, such as cancer, inflammation and immune diseases. In gastric cancers, studies reported that inducing apoptosis might reduce cell proliferation and EMT, and thus suppress gastric cancer^27,28^. And numerous studies are now focusing on drugs and new molecules such as miRNA and LncRNA that can regulate apoptosis as a target for gastric cancer treatment. However, in *H. Pylori* infection, and NSAIDs induced gastric mucosa lesion, apoptosis level was observed to be increased. And suppressing apoptosis may alleviate gastric mucosa injury^29–31^. There were also former studies^32,33^ reporting increased apoptosis in HCl/ethanol induced gastric mucosa lesion, which was consistent with our results. We also found the same effect in obesity-associated gastric mucosa injury. Furthermore, the effect of gain and loss of function of LCN2 on apoptosis suggests that the protection of LCN2 from gastric mucosa injury may act by regulating apoptosis. To verify the effect of LCN2 on regulating apoptosis, we examined the pathways of ER stress-related apoptosis. Our results revealed that the chemical induced endoplasmic reticulum stress signaling was remarkably repressed by LCN2 protein treatment. Taken together, our results suggest that LCN2 may alleviate gastric mucosa injury by inhibiting endoplasmic reticulum stress-mediated apoptosis signaling.

In conclusion, we are the first to locate LCN2 expression in parietal cells in this study. And our data revealed that the elevated LCN2 expression in obesity might be a potential protective factor against gastric injury by repressing endoplasmic reticulum stress-mediated apoptosis. The detailed mechanisms that mediate LCN2 function warrant further investigations.

## Materials and methods

### Human samples

Patients who underwent surgery in the General Surgery Department of Shanghai Tenth People’s Hospital were recruited through advertisement flyers. The study enrolled non-obese, diabetes-free patients who were scheduled for gastrointestinal surgery (subtotal gastrectomy for gastric tumor) as the control (NOB) group. Patients with morbid obesity who were scheduled for laparoscopic sleeve gastrectomy were recruited as the OB group. Obesity was defined as BMI >35 kg/m^2^ in accordance with the Asia-Pacific criteria (Bassett. 2000). These patients are newly diagnosed, with no history of medication. Normal tissue samples were collected from the stomach of the NOB group and used as control. For each patient, tissue was collected from the middle of the greater gastric curvature and part of it then further divide it into gastric mucosal layer and gastric muscular layer. All studies were approved by the ClinicalTrials.gov (ID: NCT04573998) and informed written consent was obtained from all participants or their guardians.

### Microarray analysis

Total RNA was extracted from gastric mucosa isolated from human stomach using Trizol reagent (Invitrogen). Microarray analysis was performed using 8x60K AWHGO (Agilent Whole Human Genome Oligo) according to the manufacturer’s instructions. Following hybridization, chips were scanned with a G2565CA Scanner. Data were normalized using the Mas5 method, and then log2 transformed. Micro array data can be accessed at the Supplementary Data File.

### Mice

All animal-related procedures described in this study were approved by the Animal Care and Use Committee of The Tenth People’s Hospital of Shanghai, Tongji University (ID: SHDSYY-2020-2416). All mice used in our studies were 6-8-week-old male C57BL/6 and purchased from Shanghai Laboratory Animal Center (Shanghai, China). Investigators were blinded during experiments and outcome assessment.

All animals were housed under 12:12 h light-dark conditions at 22 °C, provided with water ad libitum. Chow-fed animals were given a regular chow diet (RCD) and HFD cohort was given a diet with 60% kcal from fat (Medicience Ltd., Jiangsu, China) for 12 weeks.

#### Acute gastric mucosal injury studies

The mice were fasted for 24 h and received water ad libitum before studies. Control group were perfused with 200 ul 0.9% saline. Indomethacin group were perfused with 20 mg/ml indomethacin suspension (20 mg/kg, indomethacin tablets were dissolved in 5% sodium bicarbonate solution and diluted with purified water). Acidified ethanol group were administered through oral gavage with 150 μl of acidified ethanol (60% ethanol in 0.15 mol/L HCL). Ethanol group were given 0.01 ml/g ethanol. Mice were euthanized 4 h after administration. Stomach samples were rapidly collected, opened along the greater curvature and rinsed with ice-cold saline to remove the gastric contents in order to assess the extent of gastric damage. Gastric mucosal injury was assessed by Guth modified scoring criteria; the degree of pathological damage of gastric mucosa was scored according to Masuda criteria. Thereafter, each stomach was dichotomised, with one moiety of stomach immersed in 4% formaldehyde for histological evaluation and gastric tissue from the other moiety stored at −80 °C for biochemical determinations.

### mRNA analysis

RNA isolation, cDNA preparation and real-time PCR analyses were carried out following protocols provided by the manufactures of the kits. Trizol reagent (Invitrogen/Ambion 15596-026) was used for RNA extraction, PrimeScript^TM^ RT Reagent Kit (TaKaRa Japan) for reverse transcription PCR and SYBR Green Master Mix (KAPA Japan) for quantitative PCR. The β-actin gene was used as an internal control for gene expression analysis. TNFα and IL6 expression levels were verified using qPCR. Data are presented as fold change over control, unless otherwise indicated. Primer sequences are available upon request.

### Western blotting

Western blottings were performed as previously described^34^ using the following antibodies: anti-LCN2 (ab63929, Abcam) and anti-Caspase3 (9664 S, Cell Signaling Technology). For signal-transduction pathways, cells were lysed and analyzed by western blotting using the following antibodies: anti-Bip (3177, Cell Signaling Technology), anti-PERP (5683T, Cell Signaling Technology), anti-pPERK (3179 S, Cell Signaling Technology), anti-eIF2α (5324T, Cell Signaling Technology), anti-peIF2α (3398 T, Cell Signaling Technology), anti-CHOP (5554, Cell Signaling Technology), anti-ATF4 (sc-390063, Santa Cruz). Anti-Tubulin (T5201, Sigma) was used as an internal loading control. Experiments were repeated at least three times and representative images are presented.

### Immunofluorescence

Stomach specimens were fixed with 4% formaldehyde, embedded in paraffin, and cut into 4 um thickness tissue sections. Antibodies of LCN2, Hydrogen Potassium ATPase Beta, and Pepsinogen I were diluted by 100, 50, and 50-fold, respectively. Tissues were blocked for 30 min with 10% goat serum and permeabilized with 0.2% Trinton-100X before incubated primary antibody at 4 °C overnight. Tissue sections were washed in PBS for 3 times and followed by incubation with the FITC/TRITC-labeled secondary antibodies (Jackson ImmunoResearch Laboratory, USA) at 37 °C for 1 h. After washing again in PBS for 3 times, nuclei were stained with 49, 6-diamidino-2-pheny-lindole (DAPI) (Sigma-Aldrich, St. Louis, MO, USA) for 10 min and fluorescence analysis was performed using a confocal laser scanning microscope and Zen2011 software.

### Immunohistochemistry and tunel

Stomach sections (4 μm) were dewaxed in xylene, hydrated through an upgraded ethanol series. Antigen retrieval was achieved through boiling tissue sections in EDTA (1 mM, pH 8.0) for 15 min in a microwave oven. Endogenous peroxidase activity was quenched with 0.3% hydrogen peroxide solution for 10 min at room temperature. After washing with PBS, slides were blocked with 10% goat serum for 30 min. Slides were subsequently incubated with cleaved-caspase 3 (1:200 dilution) overnight at 4 °C. Antibody binding was detected with Peroxidase/DAB (Gene Tech, Shanghai, China). Then sections were counterstained with hematoxylin.

Stomach sections (4 μm) were dewaxed in xylene, hydrated through an upgraded ethanol series. Cell apoptosis was evaluated by using a TUNEL kit (Roche, Basel, Switzerland). TUNEL assay was carried out according to the manufacturer’s protocols.

Positive areas stained with cleaved-caspase 3 and cell apoptosis were observed in all specimens under a microscope at a magnification of ×400 by three pathologists who were unaware of specimen origins.

### Oxidative stress measurement

The activity of MPO in gastric mucosa tissue samples was determined by measuring the H_2_O_2_-dependent oxidation of tetramethylbenzidine (TMB). In its oxidized form, TMB is blue, which was measured spectrophotometrically at 655 nm. Enzymatic activities of MPO were measured according to the protocol provided by the manufacture of the kit (Nanjing Jianshe Bioengineering Research Institute). One unit of MPO activity was defined as the amount of enzyme that decomposed 1 lmol of H_2_O_2_ per min. The results were expressed as units of MPO/g tissue.

Superoxide dismutase (SOD) activities were determined also using colorimetric assay kits. Assays were performed by following the protocol provided by the manufacturer of the kit. Superoxide dismutase activity was measured by the degree of inhibition of red formazan dye formation following the reaction of superoxide radicals with 2‐(4‐iodophenyl)‐3‐(4‐nitrophenol)‐5‐phenyltetrazolium chloride that was detected at 505 nm. The results were expressed as units of SOD/g tissue.

### Cell cultures

AGS (obtained from ATCC) was maintained in Ham’s F12 (BNCC Ltd. Wuhan, China) and supplemented with 10% FBS (Gibco) and 1% penicillin-streptomycin (Gibco). GES1 (obtained from Beijing Institute for Cancer Research) was maintained in RPMI 1640 medium (Gibco, Life Technologies, Carlsbad, CA, USA) containing 10% FBS and 1%penicillin-streptomyci. All cells were incubated in a humidified atmosphere of 5% CO_2_ at 37 °C. Cell lines were tested and found to be free of mycoplasma.

Three small interfering RNAs (siRNAs) targeting human LCN2 were designed and synthesized by RiboBio (Guangzhou, China). 3 siRNAs were tested in pilot studies and the siRNA with the highest efficiency was selected for further studies. The siRNA sequences (5′–3′) was as follows: CTATGGTGTTCTTCAAGAA. LCN2 siRNAs were transfected into AGS and GES1 cells using Lipofectamine 3000 transfection reagent (Invitrogen, Carlsbad, CA, USA). Scramble siRNA was used as control and delivered in to cells using the same conditions. Real-time quantitative PCR was used to validate the efficiency of LCN2 knockdown.

### Cell viability and apoptosis measurement

AGS and GES1 were seeded in 96-well plates in 100 μl complete medium at a density of 1 × 10^5^ cells/ml. Triplicates were used in each group. The supernatant was aspirated and washed twice with PBS before chemical treatment. For the ethanol damage study, negative control cells were incubated with complete medium with no ethanol, and the other groups were treated with various concentrations (1%, 2%, 4%, 6%, 8%, 10%) of ethanol for different time points. For palmitic acid-induced toxicity study, the control cells were incubated with regular medium without additional palmitic acid, and the other groups were treated with various concentrations (100uM, 200uM, 400uM, 800uM) of Palmitic acid for different time points. At the end of the incubation, 10 μl of CCK8 solution was added into each well. Plates were kept at 37 °C for another 2 h. The absorbance was measured at 450 nm using a microplate reader. Half maximal inhibitory concentration (IC50) values and combination index (CI) were evaluated by using CalcuSyn software, Version 2.0 (CI < 1 indicates synergistic effect).

Cell apoptosis assessment was performed using the double-staining method of the Annexin-V/PI apoptosis detection kit (BD Pharmingen, Franklin Lakes, NJ, USA). AGS and GES1 cells were seeded in 6-well plates with 2 ml complete media at a density of 2.5 × 10^5^ cells/m. For LCN2 treated group, LCN2 protein (1757-LC-050, R&D Systems) was added with a final concentration at 250 ng/ml. Refer to the product manual for the detection method of LCN2 recombinant protein biological activity. To induce ethanol-mediated gastric cell damage, diluted ethanol was added at a final concentration of 6% and 4% for AGS and GES 1 cells, respectively. The treatment was for 4 h for both cell lines. For the palmitic acid induced toxicity study, palmitic acid was added at 300 μM and 400 μM for AGS and GES 1 cells, respectively, and the treatment is for 12 h.

Cell apoptosis assessment was also performed using the DAPI/PI. Cells treatment was the same as above. Then cells were collected, washed in PBS, and incubator with 49, 6-diamidino-2-pheny-lindole (DAPI) (Sigma-Aldrich, St. Louis, MO, USA) for 15 min. Then incubated with PI (Sigma-Aldrich, D9542, USA) for 5 min and fluorescence analysis was performed using microscope (CTR6000; Leica, Wetzlar, Germany).

### Statistical analysis

Results are presented as mean ± s.e.m. Unpaired, two-tailed Student’s *t* test was performed for comparisons between two groups and one-way ANOVA for comparisons of more than two groups. For all experiments *P ≤ 0.05 or #*P* ≤ 0.05. Sample-size determinations were based on the means and variances of preliminary data to achieve 80% power and a 5% experiment-wise error rate.

## Supplementary information

Supplementary information

Supplement figure

Supplementary Data File
