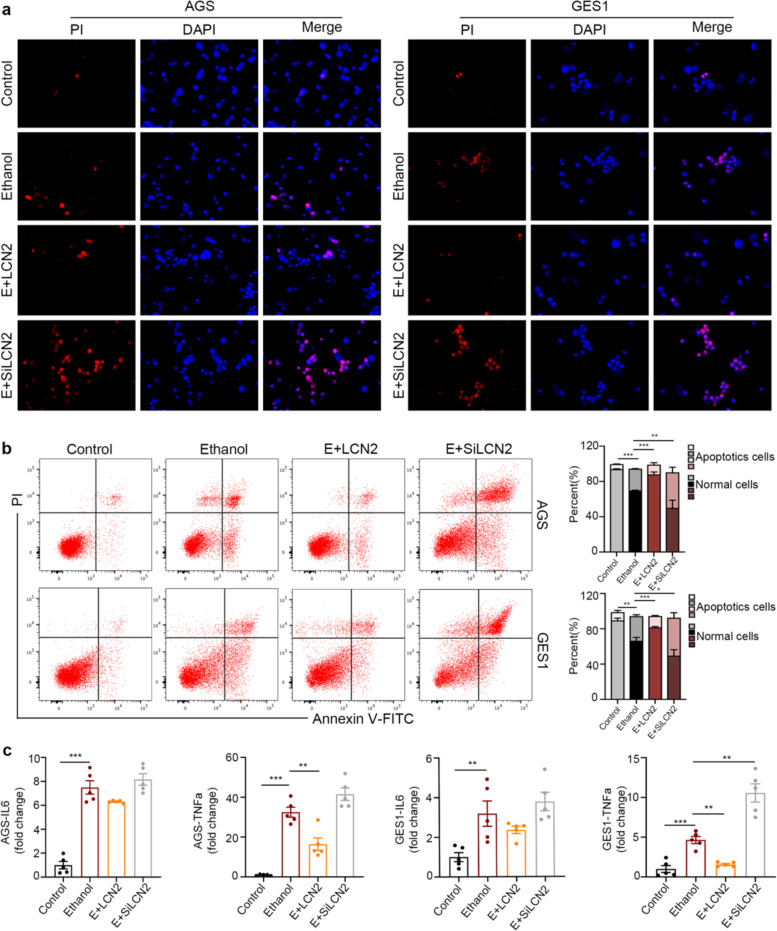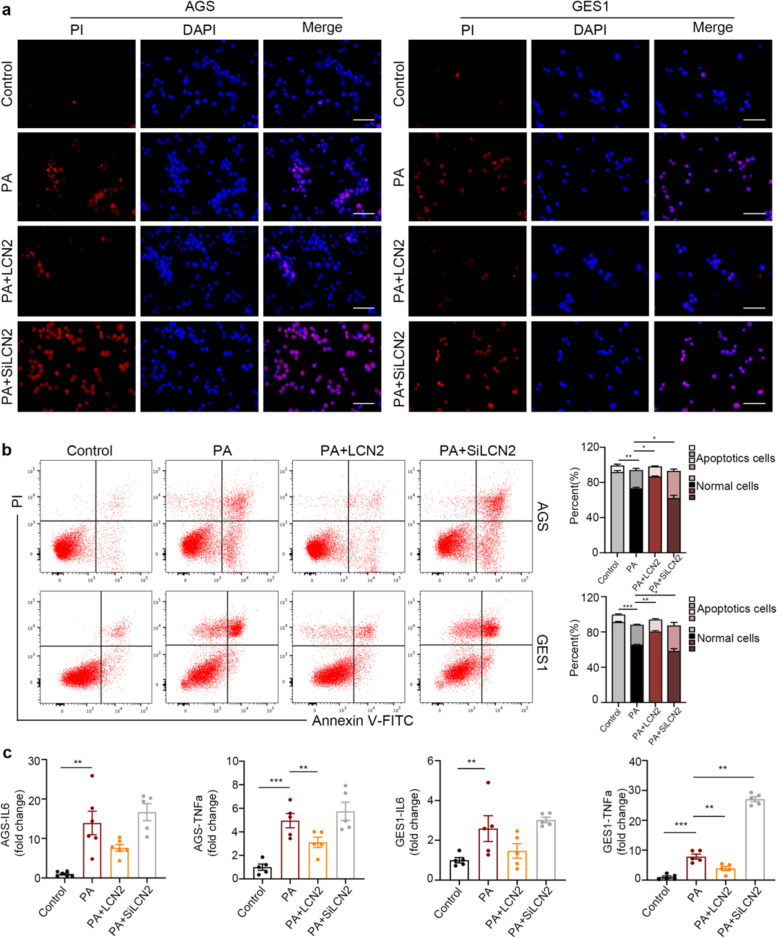# Correction to: Obesity-associated up-regulation of lipocalin 2 protects gastric mucosa cells from apoptotic cell death by reducing endoplasmic reticulum stress

**DOI:** 10.1038/s41419-022-05546-6

**Published:** 2023-03-06

**Authors:** Xin Wen, Bin Su, Mingming Gao, Jiaqi Chen, Donglei Zhou, Hui You, Nannan Li, Shuaikang Chang, Xiaoyun Cheng, Chunhua Qian, Jingyang Gao, Peng Yang, Shen Qu, Le Bu

**Affiliations:** 1 Department of Endocrinology and Metabolism, Shanghai Tenth People’s Hospital, Tongji University School of Medicine, Shanghai, 200072 China; 2 National Metabolic Management Center, Shanghai, 200072 China; 3 Department of Pharmaceutical and Biomedical Sciences, College of Pharmacy, University of Georgia, 250 West Green Street, Athens, GA 30602 USA; 4 Department of Endocrinology and Metabolism, Suzhou Municipal Hospital, The Affiliated Suzhou Hospital of Nanjing Medical University, Suzhou, China; 5 Department of Gastrointestinal Surgery, Shanghai Tenth People’s Hospital, Tongji University School of Medicine, Shanghai, 200072 China; 6 Department of Hematology, Shanghai Tenth People’s Hospital, Tongji University School of Medicine, Shanghai, 200072 China

**Keywords:** Apoptosis, Stomach diseases

Correction to: *Cell Death and Disease* 10.1038/s41419-021-03512-2, published online 26 February 2021

The original version of this article contained errors in Figs. 4b and 5b. In Figs. 4b and 5b, the authors showed the representative images for flow cytometry on gastric epithelial cells (AGS, GES1) stimulated by Ethanol and PA. These two figures confirmed each other and proved similar results suggesting that LCN2 protects gastric epithelial cells from apoptosis. Recently the authors found that Figs. 4b and 5b are the same in the final published version. They have checked all the raw data and reviewed their previous submission histories. This mistake was generated during submission. The authors sincerely apologize for this mistake. The correct figures can be found below. The original article has been corrected.