# Supplementary material for: Obesity-associated up-regulation of lipocalin 2 protects gastric mucosa cells from apoptotic cell death by reducing endoplasmic reticulum stress
Source: Cell Death Dis. 2021 Feb 26;12(2):221. doi: 10.1038/s41419-021-03512-2 (PMC7910621; doi:10.1038/s41419-021-03512-2)
Supplement: Supplementary file 1 — Supplementary information [file 41419_2021_3512_MOESM1_ESM.docx]

**Obesity-associated Up-regulation of Lipocalin 2 Protects Gastric Mucosa Cells from Apoptotic Cell Death by Reducing** **Endoplasmic Reticulum Stress**

Xin Wen^1,2#^, Bin Su^1,2#^, Mingming Gao^3^, Jiaqi Chen^1,4^, Donglei Zhou^5^, Hui You^1,2^, Nannan Li^1,2^, Shuaikang Chang^6^, Xiaoyun Cheng^1,2^, Chunhua Qian^1,2^, Jingyang Gao^1,2^, Peng Yang^1,2^,Shen Qu^1,2*^, Le Bu^1,2*^†

**Supplement Fig. Selection of optimal stimulus conditions.** AGS and GES1 cells were treated with ethanol (0%, 2%, 4%, 6%, 8%,10%) for different times (2h, 4h, 6h) or palmitic acid (0mM, 0.1mM, 0.2mM, 0.4mM, 0.8mM) for different times (12h, 24h), and cell viability was analyzed using a CCK8 kit. Then IC50 of cells after stimulated were determined by using CalcuSyn software, Version 2.0.
