# Supplementary figures and images for: Obesity-associated up-regulation of lipocalin 2 protects gastric mucosa cells from apoptotic cell death by reducing endoplasmic reticulum stress
Source: Cell Death Dis. 2021 Feb 26;12(2):221. doi: 10.1038/s41419-021-03512-2 (PMC7910621; doi:10.1038/s41419-021-03512-2)

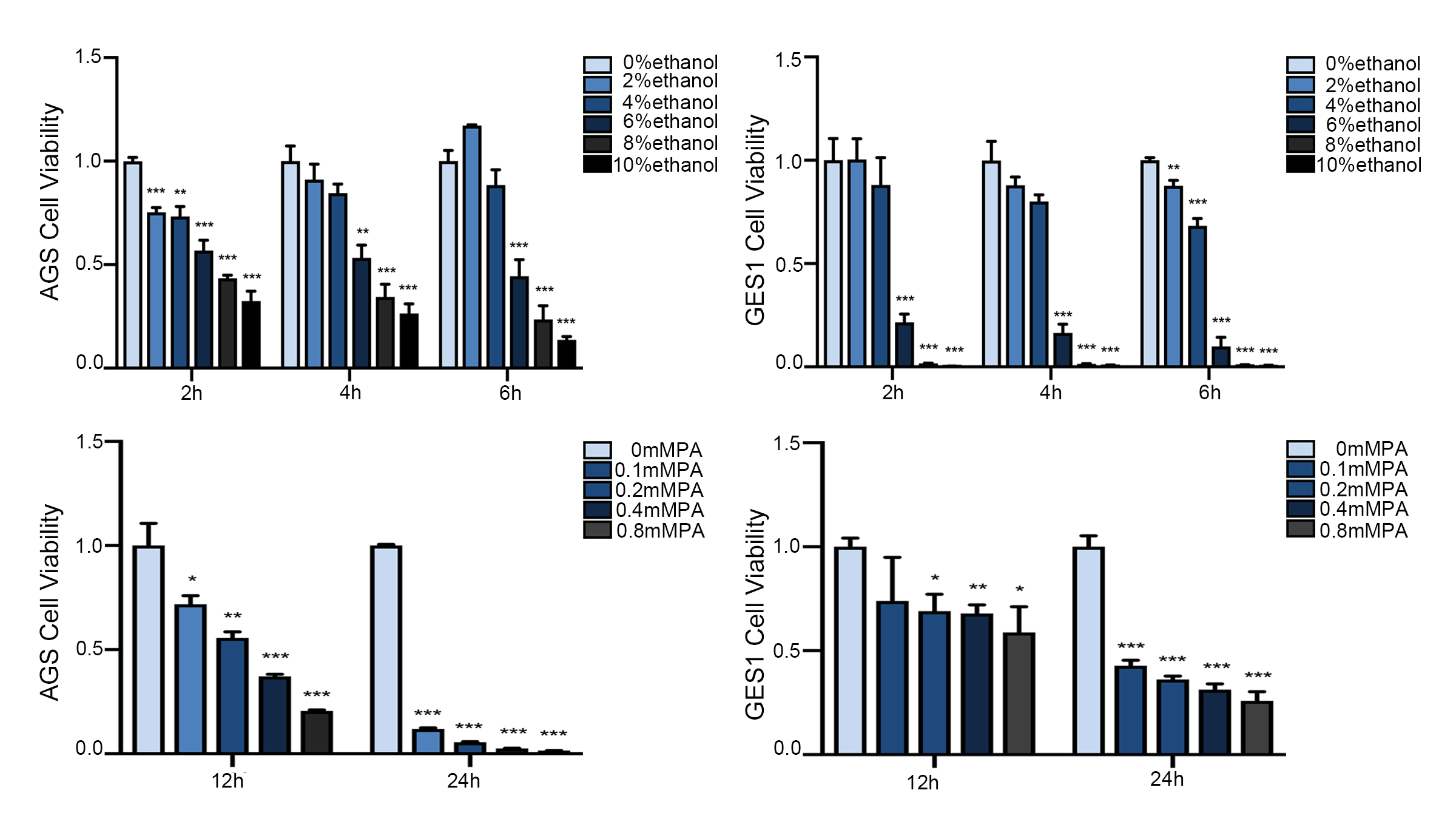

Supplement: Supplementary file 2 — Supplement figure [file 41419_2021_3512_MOESM2_ESM.tif]
